# Not so once upon a time

**DOI:** 10.1016/j.rpth.2023.102288

**Published:** 2023-11-30

**Authors:** A. Valance Washington

**Affiliations:** Department of Biology, Oakland University, Rochester, Michigan, USA

**Keywords:** discrimination, intolerance, psychological resilience, racism, sexism

## Abstract

This story reflects a personal narrative of a graduate student who experienced a disturbing incident during Mardi Gras in New Orleans, shedding light on the enduring issue of racism. The author's journey to graduate school and the challenges faced along the way provide context for the pivotal moment of racial discrimination. The narrative then shifts to the author's decision to prioritize education over anger, highlighting the sacrifices made to protect their future as a graduate student. The incident serves as a stark reminder that, despite personal achievements and aspirations, racial prejudice persists. In conclusion, the author calls for resilience and focus in the pursuit of personal goals while acknowledging the ongoing struggle against racism and other forms of discrimination in society. This personal story serves as a poignant reminder of the challenges faced by individuals and the need for continued efforts to combat systemic intolerance.

I was marched around the corner and asked for my identification in front of a small bus. The bus looked like one of the very short public transport vehicles. It was dark. Minimal illumination from the nearby streetlight reached the spot where we were standing. The officer looked at my identification, handed it back, and commanded me to get on the bus. Three other policemen surrounded me in silence.

Graduate school, at least for me, was an education beyond my class. It was a time of making lifelong friends, self-discovery, and maturation. But much like testing gravity by parachuting, I have also learned things that should have never been part of my college or life curriculum.

As far as I can remember, hard work was always part of my upbringing. Its value was instilled in me by the example of my parents, who took pride in earning what they had. From selling flower seeds door-to-door to having my own paper route and serving customers at local food establishments, my experiences taught me aspiration and resilience. After getting my undergraduate degree, I worked for 6 years in corporate America, building a stable career. Yet, I felt drawn to another path. I wanted to do more. To be more. Earning a PhD was the path I chose to follow.

I decided on a school in Dallas, Texas. My 6-year outdated Graduate Record Exam allowed me to be accepted and receive a tuition reduction but did not help me get a teaching assistantship. I had to provide for my room and board, but I still saw this as an opportunity. So, I resigned from my job, packed my things, and moved to Dallas.

The first semester fully tested my resilience. I managed to get a job at Radio Shack, which was flexible to my school schedule and paid me enough to get by, that is, if I didn’t own a car or visit any local watering establishments. Yet, it was taking time away from studying. For months, I lived on 2-3 hours of sleep: a class at 8 am, the laboratory from noon to 4 pm, then work until 9:30 pm. At home, I would eat and study past 2 am, only to wake up to repeat the same routine. I learned to focus and manage my time, and I passed all classes, earning my teaching assistantship for the next semester.

From there, things began to look up. I joined a laboratory that studied drosophila genetics. As the student graduate representative, I became a hall director, which gave me housing and an additional stipend. I joined the local rugby club. I made friends, not just in biology but throughout the university community. Together, we worked hard, learned about Dallas culture, and, over the years, watched the city blossom into a busy metropolis. It was a great time to be in Dallas.

The idea of going to New Orleans, Louisiana, for Mardi Gras came as an unexpected opportunity. A friend, a senior premedical student, and New Orleans native, had just purchased a new Saturn and was eager to show it off on a 7-hour drive to the city. There were 4 of us on this trip: in addition to me and the driver, there was a residence life coordinator and a colleague of the driver. We were all staying with our New Orleans friend’s parents – a perfect solution for a graduate student budget.

We set off on a lovely Friday afternoon and arrived in time to enjoy the evening's festivities. After stopping at the house for a quick meal, we headed to Bourbon Street. It was dusk. I knew that Mardi Gras was one of those legendary gatherings akin to New Year’s Eve in New York City or the Full Moon Party in Koh Phangan. Oddly enough, even though I grew up in the suburbs of New York, I had never attended the New Year’s Eve celebration. So, nothing I had done to this point in my life prepared me for what I saw.

The crowd was unreal, alive. Standing on a balcony, looking down, I watched the crowd pulsate like a heartbeat. It seemed to swell, then shrink and swell again—An organized chaos. Within the crowd, thousands of little lives were doing their own little things. Some were moving up the street while others were moving down. Groups of people were standing along the edges; some stood in the middle of the road, and the crowds walked around them. Crossing the street in a straight line was impossible. It would have been easier to swim across the Mississippi.

And there we were, walking at the fringes of the crowd to our next destination. We walked a single line between those standing against the establishments and those meandering in the street. Our leader had a plan: we were going to visit friends, so our movement through the crowd was more directed than most. The crowd pulsated as we moved in and out. My toes were stepped on a thousand times, but I didn’t mind it at all since I too, accidentally stepped on more than one toe. In most instances, people were too busy to notice; other times, they replied with “No worries; it is Mardi Gras” when I stopped to apologize.

I stepped on one toe, however, that put things in an entirely different perspective. I was following my friend's single line through the crowd when I felt my foot land on someone else’s. Smiling apologetically, I turned around to a look I would never forget. Anger was spreading across a man’s face and growing into rage. “I will teach you what it is to be sorry!” he barked, piercing me with his eyes. I stood motionless – this person was an officer of the law.

My friends would not see me for the rest of that evening. He told me to get on the bus as the other 3 officers surrounded me in silence. I had no choice. I walked up the stairs. There were 2 people arrested for urinating in public. They sat quietly, deflated, and intoxicated. I could not wrap my mind around this. What was I doing there?

Just as I asked the officers why I was there, I felt a dull thud across my back. A wave of pain was followed by another thud. And then one after the other. Instinctively, I covered my head for some protection, and then I heard the words: “If you think Rodney King was beaten badly…” It was then that I realized why I was there: it was not for *what* I did wrong; it was for *why* I was wrong. I was black. The last thing I remember was everything fading to darkness while I was trying to reach for my license, now on the floor. The thuds on my back were getting duller until I felt no more.

I regained consciousness the next morning to the smell of urine and alcohol saturating a jail-holding cell. I was lying on my back, staring at a key lime green concrete ceiling. People were stepping over me. There were about 2 dozen of us; some sitting along the wall, some pacing back and forth. My head felt like I had 2 of them, one inside the other. I cannot recall the extent of the pain, but I remember the reflection in the acrylic bulletproof glass: a distorted face with one side badly swollen and an eye in a new shade of night.

I was torn somewhere between anger and fear. How could this happen to me? What did I do to deserve this? I wanted retribution. I wanted those officers to pay; I wanted the city to pay. Despite that, I had to be back at school on Monday. I could not let anyone know about my arrest and face the possibility of losing my spot as a graduate student. I had worked too hard to follow my anger. I chose my education over my indignation and rage. This was a hard pill to swallow, a pill wrapped in a story of a rugby tournament brawl and getting hit in the eye.

I was lucky. My friend from New Orleans was able to pull some strings, and I was let out with a $50 fine and a sizable donation to the judge’s campaign. What I saved by staying at a friend's house, I paid to be legally assaulted. In retrospect, I *was* lucky. George Floyd did not wake up at all.

Just like for many of my peers for me, graduate school was a time of hard work and scientific exploration. I learned how to ask and answer questions in the context of scientific reason. Yet, what I learned that night was beyond reason: regardless of my work, education, or personal qualities, I was still a black male.

Over the years, I have come to realize that, sadly, my experiences as a black male in America were not very different from those of other groups that fall outside of the default. Any social default in its core is a moral defect, leading to racism, sexism, bullying, intolerance, bias, and prejudice. Intergroup conflict – a constant companion of human existence – stems from a disproportionate amount of power given to dominant groups, marginalizing many people within a country or society. Girls and women, people of color, religious communities, citizens in poverty, LGBTQ people, youth, and children—all have endured the consequences of the default.

That being said, there is still a very small percentage of males in science, technology, engineering and mathematics (STEM) fields, pointing to added social pressures that we face. Many black males do not have 2 parental models. Often, the black male role model is missing. I was fortunate to have 2 strong role models in my parents. I have always fought to be a role model in the lives of my children. The hardest battles to fight were those that were institutionalized. As much as I try to bury what happened to me in New Orleans, the reoccurrence of such stories as George Floyd, Tamir Rice, Eric Garner, and many others remains a constant reminder that I cannot give up that fight and these events are not so once upon a time.

For the last 2 decades, our society has been moving toward eradicating racism. What happened to me a few years after the Rodney King incident was over a quarter of century ago. It should have been a thing of the past, but to some extent, it seems worse today than in years past. For as long as we as a society continue designating a particular alternative as the default, nothing will move this needle. What I can say is—be resilient, remember your personal goals, stay focused on them, and continue moving onward and upward toward your own light.

